# Supplementary figures and images for: In vivo recellularization of xenogeneic vascular grafts decellularized with high hydrostatic pressure method in a porcine carotid arterial interpose model
Source: PLoS One. 2021 Jul 22;16(7):e0254160. doi: 10.1371/journal.pone.0254160 (PMC8297896; doi:10.1371/journal.pone.0254160)

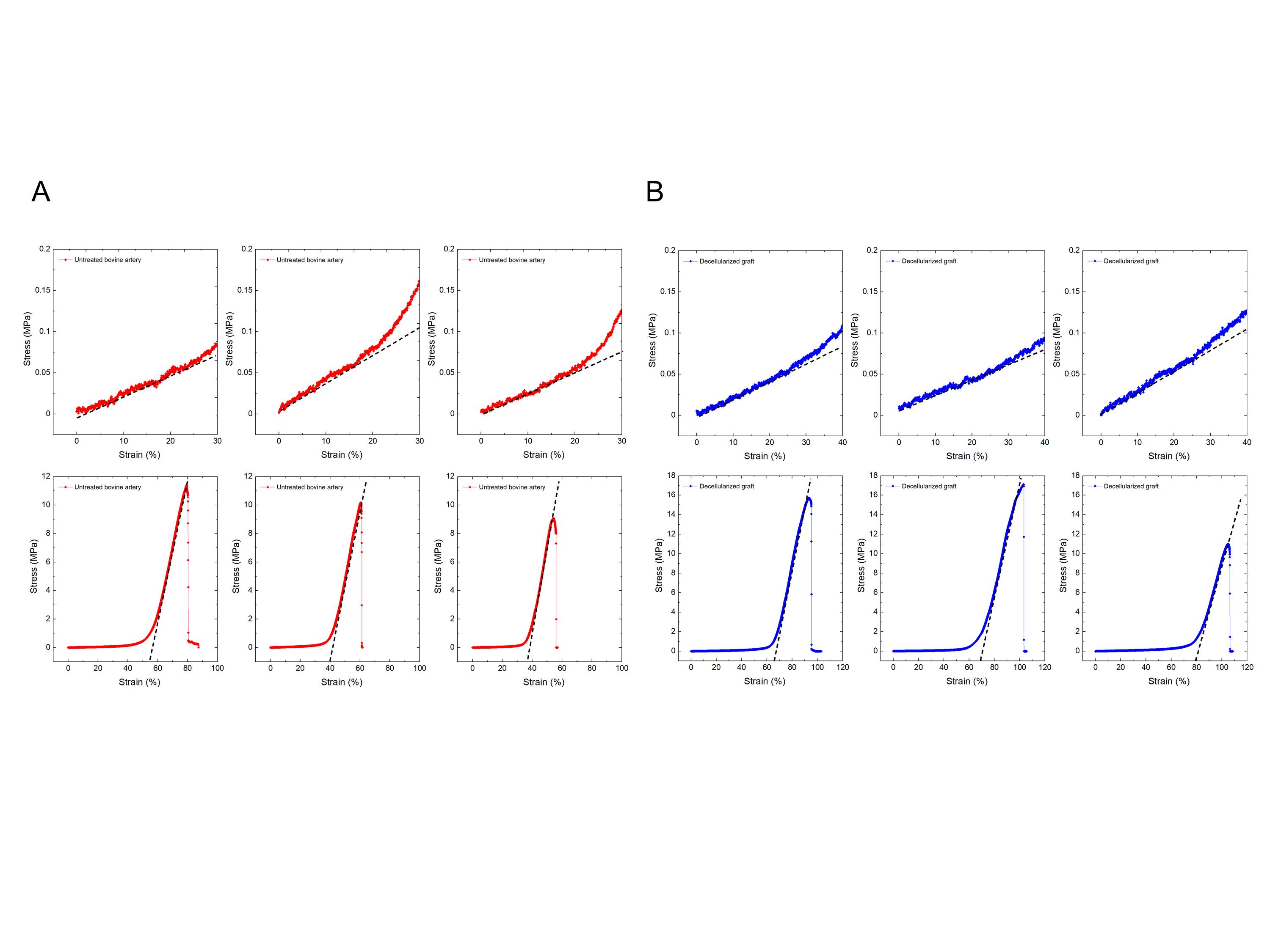

Supplement: S1 Fig — Curves between stress and strain in untreated bovine arteries (A) and decellularized arteries (B) at early phase (upper) and late phase (lower). Results of 3 independent experiments are shown. (TIF) [file pone.0254160.s001.TIF]

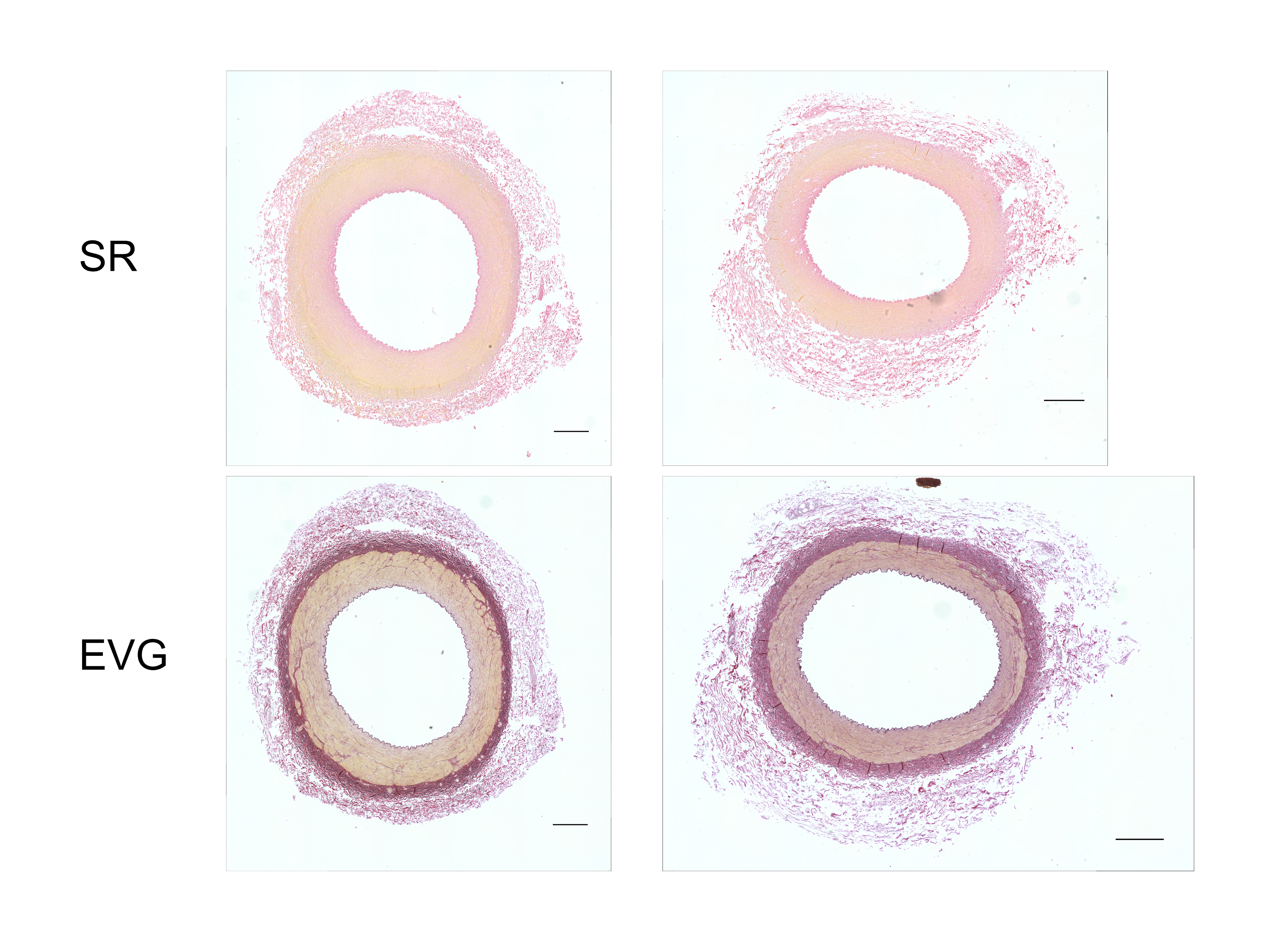

Supplement: S2 Fig — Scale bars = 500 μm. (TIF) [file pone.0254160.s002.TIF]

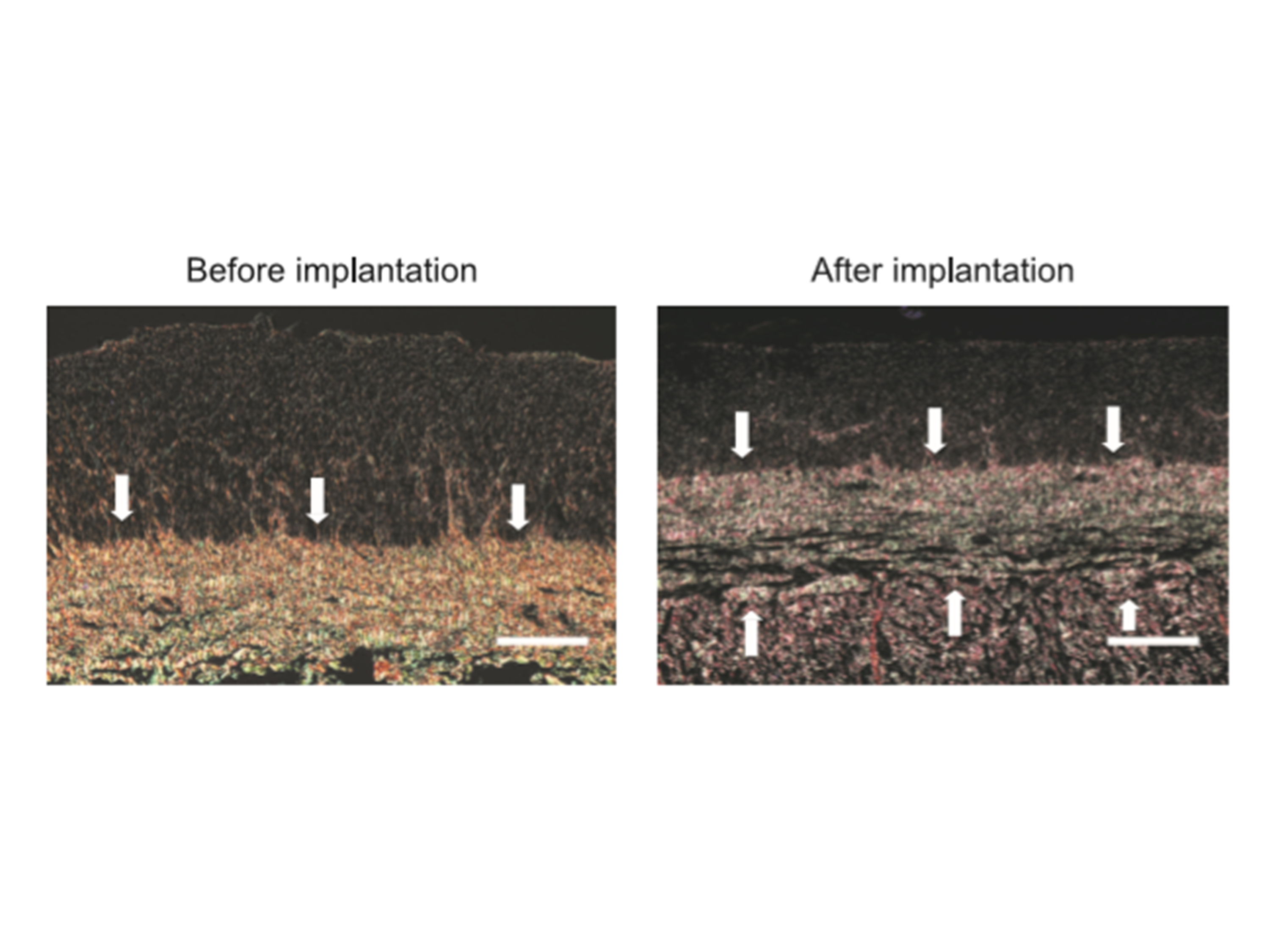

Supplement: S3 Fig — Before (left) and after (right) implantation. Scale bars = 200 μm. (TIF) [file pone.0254160.s003.TIF]

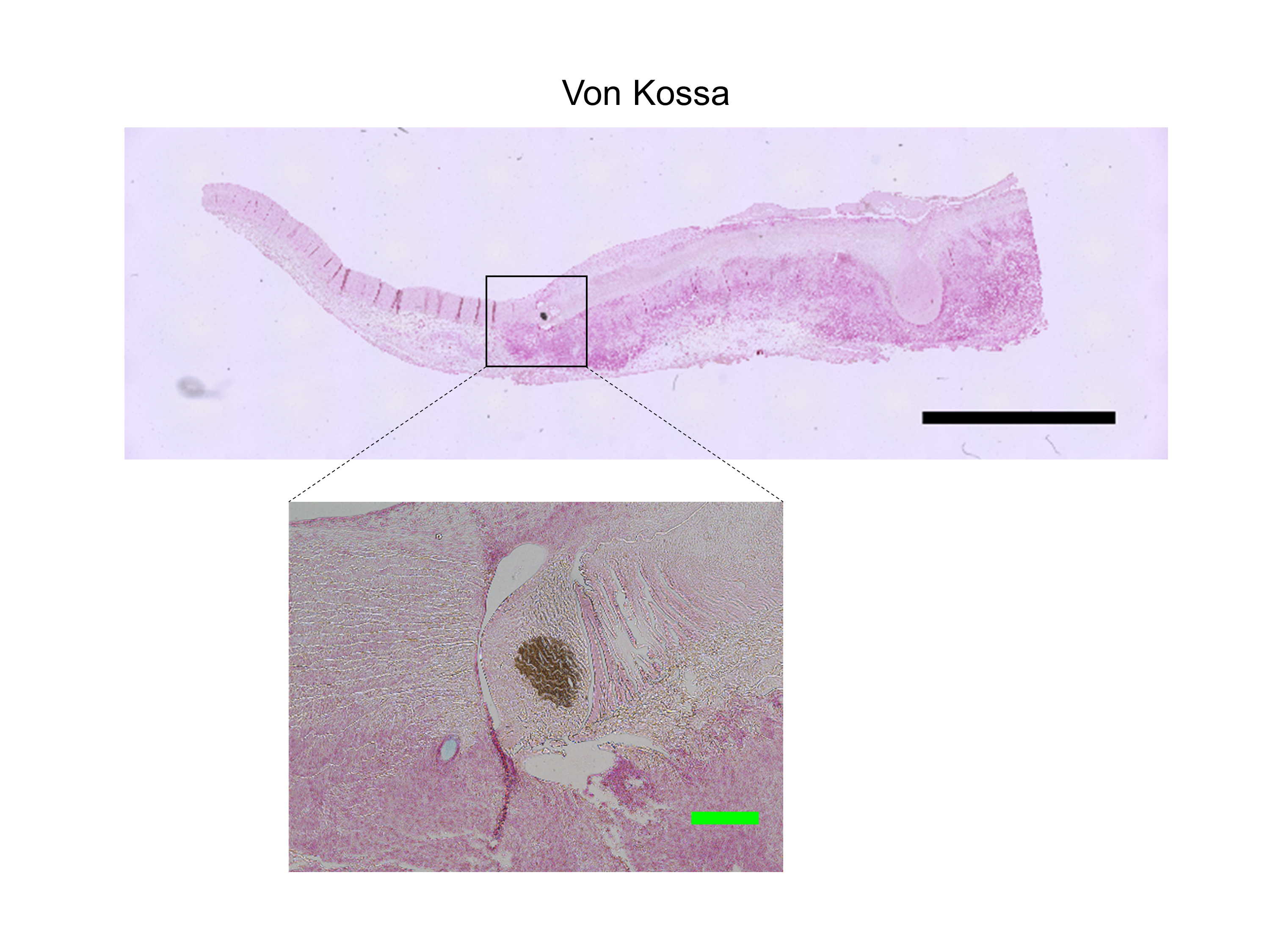

Supplement: S4 Fig — Scale bars = 5 mm (top), 100 μm (bottom). (TIF) [file pone.0254160.s004.TIF]

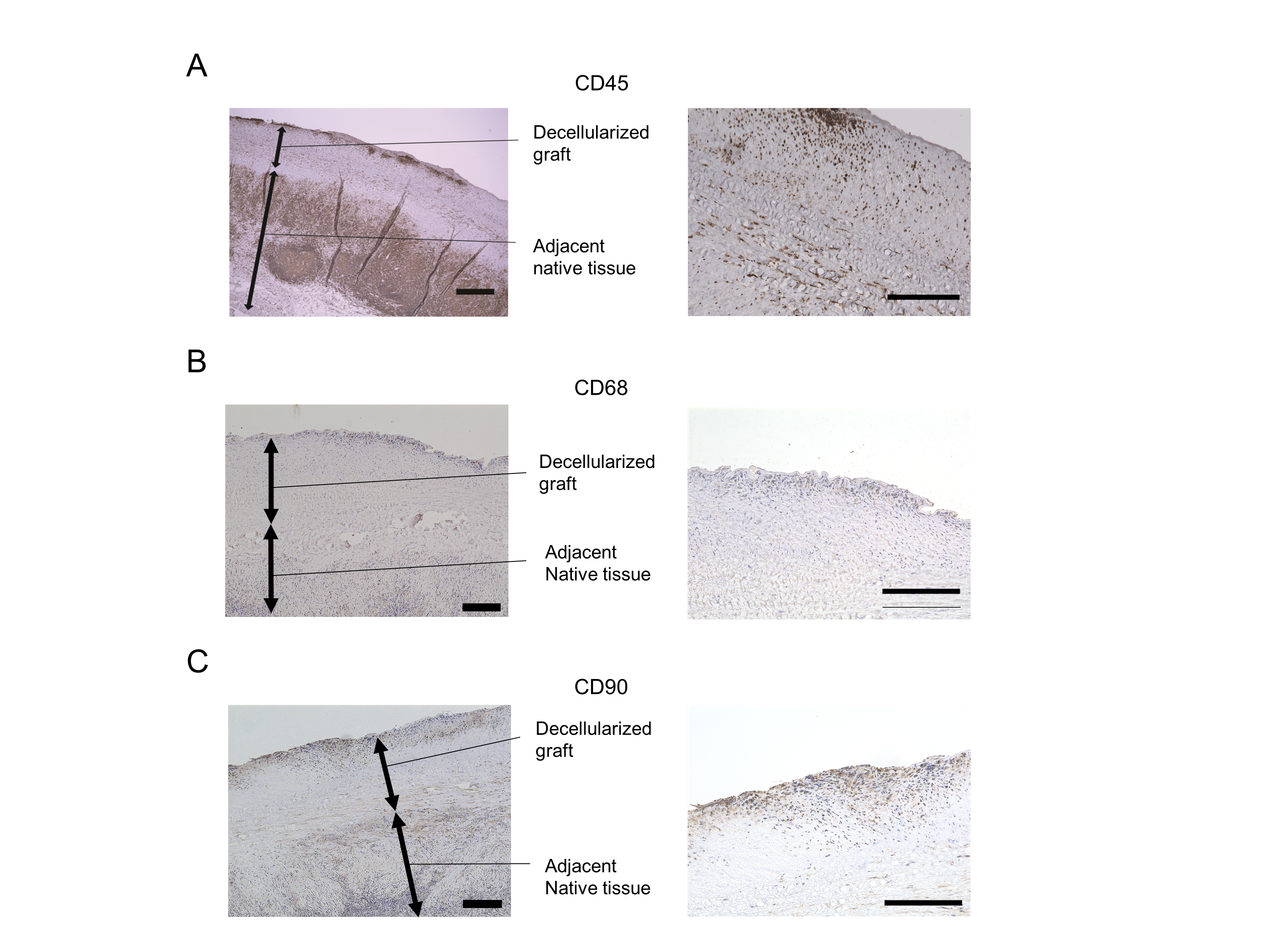

Supplement: S5 Fig — CD45 (A), CD68 (B) and CD90 (C) immunostaining for grafts after implantation. Left: lower magnification, right: higher magnification. Scale bars = 200 μm. (TIF) [file pone.0254160.s005.TIF]
