# Supplementary material for: In vivo recellularization of xenogeneic vascular grafts decellularized with high hydrostatic pressure method in a porcine carotid arterial interpose model
Source: PLoS One. 2021 Jul 22;16(7):e0254160. doi: 10.1371/journal.pone.0254160 (PMC8297896; doi:10.1371/journal.pone.0254160)
Supplement: Attachment — Submitted filename: Response to Reviewers_R1_200930.pdf [file pone.0254160.s008.pdf]

Thank you for your detailed comments and useful suggestions that have helped us to considerably improve our manuscript. Taking your comments into careful consideration, we conducted additional experiments and revised our manuscript. As indicated in the responses that follow, we have taken each of these comments and suggestions into account in the revised version of our manuscript. Modified or added sentences are highlighted in red in the main manuscript.

For Reviewer #1:

Comment #1:

Characterization of decellularization was dependent on quantification of DNA content. As this method did not introduce detergent to remove protein components, it will be good if the author can test the presence of potential remnants of surface or cytoplasmic proteins, such as surface antigen or beta actin.

Response:

We appreciate the comments. We apologize that we could not sufficiently mention our methods for decellularization. As described in MATERIALS AND METHODS section, we apply washing process by continuous gradual shaking in saline supplemented with 0.2 mg/mL of DNase I and 50 mM of magnesium chloride for 7 days and then in 80 % ethanol for 3 days after hydrostatic pressure loading (line 99 – 107, page 7). We suppose that not only DNA contents but also protein components are somehow washed out (cellular components are washed out and off course wash-outed cells include protein components). However, we also confirmed that proteins which compose tunica media of vascular tissues are preserved which was confirmed by histological evaluations (new Figure 4) and mechanical properties are preserved by the decellularization (Figure 1; new Supplemental Figure 1).

To further clarify this point, we added descriptions regarding this point in DISCUSSION section (line 350 – 351, page 20).

Major Comment #2:

It is highly recommended that the authors evaluate the amount of collagen and elastin before and after decellularization.

Response:

Thank you for the comment. According to the comment, we evaluated the amount of collagen and elastin with Sirius-red staining and Elastica van Gieson staining before

and after decellularization, respectively. We found that collagen and elastin deposition are preserved at tunica media through the decellularization procedure.

We added descriptions regarding this point in RESULTS section (line 268 - 269, page 16). We added figures of Sirius-red staining and Elastica van Gieson staining before decellularization at Figure 4.

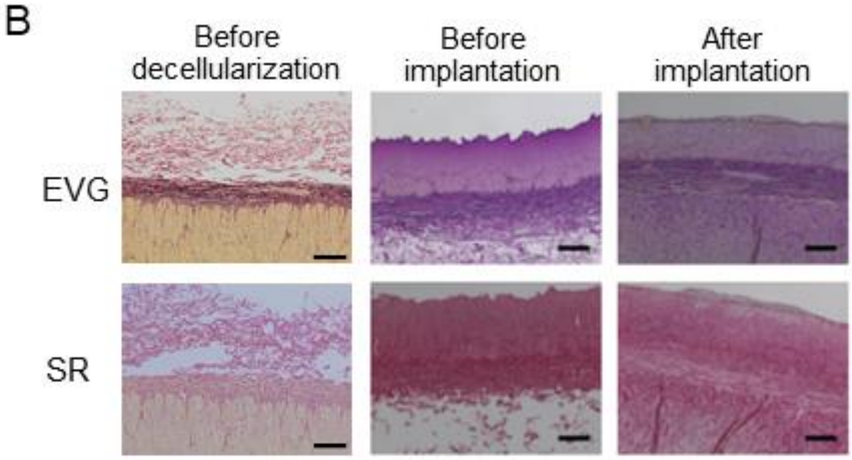

(New Figure 4B)

Major Comment #3:

When testing the tensile strength of the tissue, the authors mentioned that "The samples were cut into dumbbell shape with a length of 35 mm and a width of 2 mm." Please describe how the sectioning was performed. As a comprehensive analysis, it is important to evaluate the tensile strength of sections based on both longitudinal and circumferential sectioning.

Response:

Thank you for the valuable comments. We originally planned to prepare samples of longitudinal and circumferential directions for tensile strength evaluations. However, we found that it was impossible to prepare circumferential samples because of the rather small size of the vessels as tensile tests require certain length of the samples.

We added descriptions regarding this point in MATERIALS AND METHODS section (line 125 – 126, page 8).

Major Comment #4:

More evaluation of the explants should be done. Since this is a xenograft, so it is critical to evaluate the immunoresponse such as the infiltration of macrophages. Also the infiltration of other cell types, such as the vimentin or CD90 positive fibroblasts, should be evaluated.

Response:

We appreciate the comments. According to the reviewer's comments, we performed CD90 immunostaining for the explant. As shown in new Supplemental Figure 3B, we could confirm the accumulation of CD90-positive fibroblasts around the implanted grafts and at the luminal surface of the grafts but the fibroblasts did not infiltrate inside of the grafts which is the similar pattern of than in CD45 immunostaining.

We added descriptions regarding this point in RESULTS section (line 289, 290, page 17). We added an immunostaining image at Supplemental Figure 3 (new Supplemental Figure 4B).

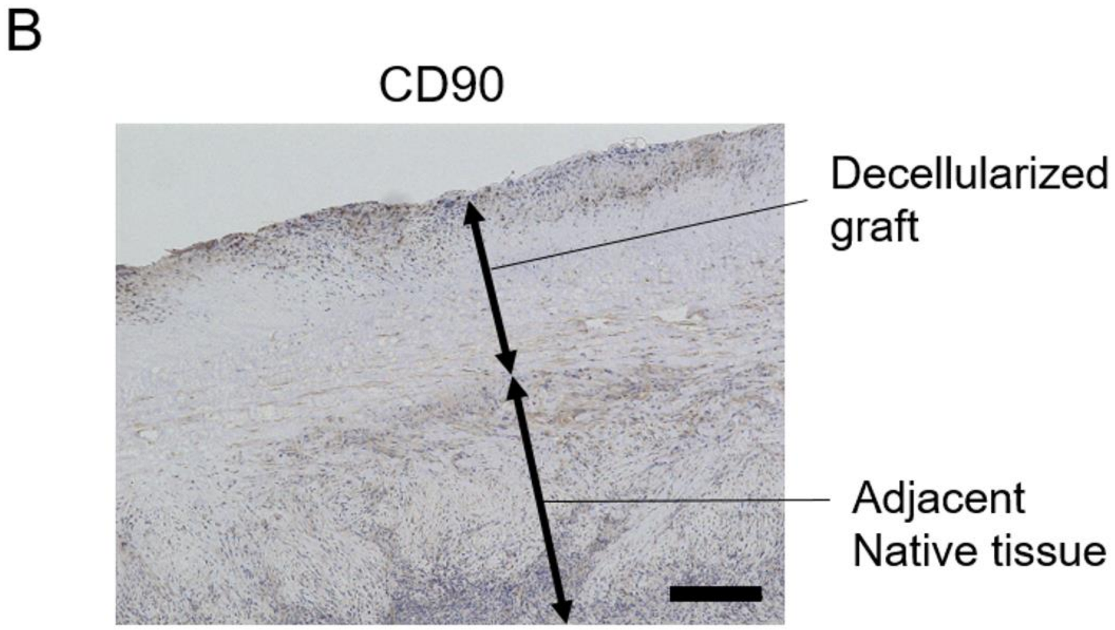

(New Supplemental Figure 4B)

<u>For Reviewer #2:</u>

Comment #1:

Firstly, can the authors provide mechanical properties information beyond just the elasticity?

Response:

We genuinely appreciate your comment. According to the comment, we additionally showed a representative stress-strain curve (new Supplemental Figure 1) in addition of the modulus of elasticity (Figure 1C,D). The patterns of the curves were almost similar between untreated and decellularized grafts even though the tensile strength (peak-top)

and elongation tended to be higher in decellularized grafts.

We added descriptions regarding this point in RESULTS section (line 213 – 215, page 13). We added curves between stroke and applied pressure as new Supplemental Figure 1.

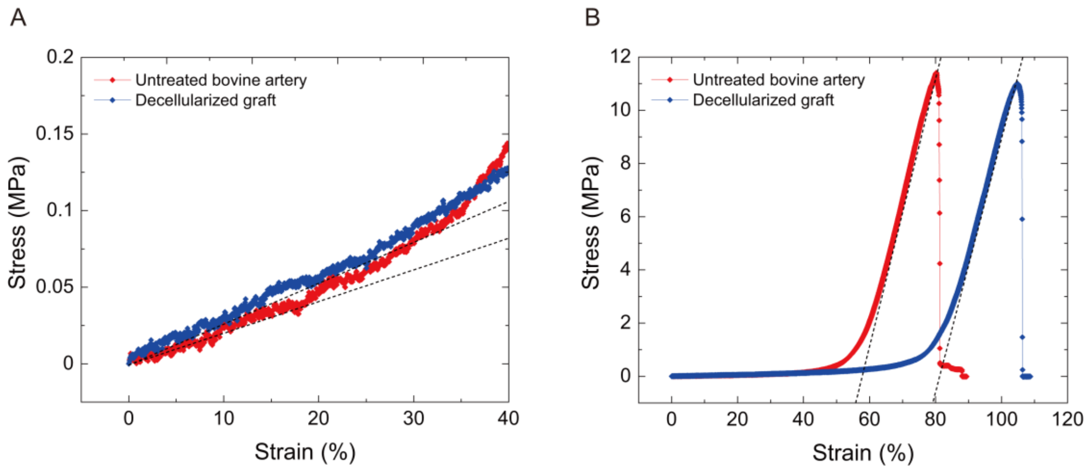

(New Supplemental Figure 1)

Comment #2:

Secondly, distal graft stenosis at 4 weeks of ~50% suggests that the grafts will eventually fail from restenosis due to intimal hyperplasia of smooth muscle cells. Therefore, can the authors be more clear about the advantages of using this grafting material? The authors state that their results indicate the feasibility of their graft. But the data suggests that the grafts will fail within just a few months.

Response:

We appreciate the comments. As the reviewer pointed out, we cannot completely abolish the possibility that the grafts would occlude in a few months.

The main concern for the occlusion would be the progression of the intimal hyperplasia which were observed in the present study (Figure 4E). It is crucial to check whether the intimal hyperplasia progresses at longer observation period which should be done in our future study.

We also need to think about the stenosis sites. Considering that the stenotic sites existed in close proximity of suture lines, there would be a possibility that turbulence of the blood flow at suture lines or the existence of suture threads affected to the stenosis formation. The establishment of preventive strategies for the stenosis is crucial towards clinical implementation of the HHP-decellularized grafts as an allogeneic graft source in the future.

We added descriptions regarding this point in DISCUSSION section as our important future direction and clarified this point as an important limitation of the present study (line 366 -367, 368 - 369, 370 – 375, page 21).

We believe that we have adequately addressed all the concerns. We look forward to receiving the next review comments.

Best regards,
Hidetoshi Masumoto, MD, PhD
